# Co-creating an intervention promoting healthy weight development during infancy

**DOI:** 10.1093/eurpub/ckac131.439

**Published:** 2022-10-25

**Authors:** L Kierkegaard, R Rothkegel Carlsson, L Ayoe Sparvath Brautsch, C Thørring Bonnesen

**Affiliations:** National Institute of Public Health, Copenhagen, Denmark

## Abstract

**Background:**

Childhood obesity is a major public health challenge, and it is recommended to promote healthy weight development already during infancy. It is important to co-create interventions to maximize the feasibility and thus improve the chances of successful implementation. This paper describes the co-creation process of the Danish Bloom Trial - an early intervention to promote healthy weight development among children of first-time parents.

**Methods:**

Development of the trial is inspired by co-creation frameworks and the Intervention Mapping protocol. The co-creation process comprises three stages: 1) Evidence review, qualitative research with community health nurses (CHNs) and parents, and stakeholder consultations; 2) co-creation of the intervention content including workshops and group meetings with CHNs and other stakeholders and focus group discussions with parents; and 3) prototyping, feasibility- and pilot-testing. Currently, we are in stage 2 and have conducted four workshops with CHNs and one parent group discussion.

**Results:**

During stage 1, we identified the intervention setting; the unique system of CHNs in Danish municipalities. Furthermore, we identified the need for developing intervention content focusing on nutrition, physical activity, sleep, screen time and sense of security to promote healthy child weight development. The main intervention components are a course for CHNs and guidelines on how to talk to parents about behavioral risk factors. The main components for parents are eight home visits and six telephone consultations from CHNs during pregnancy and until the child is 2½ years old and a video library.

**Conclusions:**

The description of the development of the Bloom Trial provides an example of how to co-create an intervention balancing evidence, the practical work of the implementers and the needs of the families. Co-creation with relevant stakeholders increases the chances of producing a relevant, successful, and sustainable intervention.

**Key messages:**

• The co-creation process resulted in development of intervention content focusing on nutrition, physical activity, sleep, screen time and sense of security from pregnancy to child age 2½ years.

• Involving parents and stakeholders in the development of an intervention increases the chances of producing a relevant, successful, and sustainable intervention.

